# The Validation of Direct and Meta Versions of the Coach–Athlete Relationship Questionnaire (ArCART-Q) in the Arabic Language: Their Relationship to Athlete’s Satisfaction with Individual Performance

**DOI:** 10.3390/ijerph18041998

**Published:** 2021-02-19

**Authors:** Hasan Ahmad, Elif Nilay Ada, Sophia Jowett, Kholoud Alabduljader, Zişan Kazak

**Affiliations:** 1 The College of Basic Education, The Public Authority for Applied Education and Training, Al Adiliya Area, Kuwait City 13092, Kuwait; Kholoud911@gmail.com; 2 Faculty of Sport Sciences, Mersin University, Mersin 33343, Turkey; elifnilayada@gmail.com; 3 School of Sport, Exercise and Health Sciences, Loughborough University, Loughborough LE11 3TT, UK; S.Jowett@lboro.ac.uk; 4 Faculty of Sport Sciences, Ege University, Bornova, Izmir 35040, Turkey; f.zisan.kazak@gmail.com

**Keywords:** coach–athlete relationship, validation, satisfaction

## Abstract

**Background**: The first aim of this study is to achieve validation of the direct and meta-perspective versions of the Coach–Athlete Relationship Questionnaire in the Arabic language, and the second aim of this study is to determine the quality of the coach-athlete relationship to athlete’ satisfaction with individual performance according to sport participation type, sport duration, and sport achievement. **Methods**: A total of 259 athletes with a mean age of 22 years were recruited from various athletic clubs in Kuwait. Participants completed The Coach–Athlete Relationship Questionnaire and The Athlete Satisfaction Questionnaire. For this study, the factorial structure of the Arabic version of the Coach–Athlete Relationship Questionnaire (CART-Q) was used in Kuwait and was prepared with both direct and meta perspectives. **Results**: The results of this study show evidence of the validity of the direct and meta-perspective Arabic versions of the CART-Q. The fit indices of the data collected by direct-method were as follows (x^2^/df = 2.21; RMSEA = 0.06; CFI = 0.98; GFI = 0.95; AGFI = 0.91); data for the meta-method were as follows (x^2^/df = 2.32; RMSEA = 0.08; CFI = 0.99; GFI = 0.93; AGFI = 0.87). Female participants have obtained higher means than males from all questionnaires. **Conclusions**: The results of the present study could help coaches and athletes from the Middle East to understand the reasons and methods that lead to a quality coach–athlete relationship.

## 1. Introduction

There are different social (e.g., coach–administrator, athlete–athlete) and personal relationships (e.g., athlete–parent, athlete–partner) that can be found in the sport context, however, the coach–athlete relationship is the most important for both performance accomplishments and psychological well-being [1,2]. While coaches need to ensure that they are creating an environment that allows athletes to feel open, accessible, and available (as opposed to withdrawn, hostile, and distant) [3], athletes find it difficult to produce top-level performances without the support of their coaches [4]. Moreover, coaches are unlikely to be successful without athletes’ talent, passion, and enthusiasm [5]. In many cases, neither the coach nor the athlete can do it alone [6]. Coaches may have to deliberately create situations that provide opportunities to connect with the athlete and create an environment that is genuinely and constantly nurturing, supportive, and caring [7]. Athletes reach out to their coaches for their expertise and knowledge, and as a result often have to set aside the sort of insecurities that are likely to prevent them from building a close, trustworthy, and committed relationship if they are to develop and succeed in sport [8].

Sports coaches encourage new skills and challenges, even in the face of adversity, and provide a platform for growth and development [9]. Through a series of studies [10,11], it was found that coaches were viewed by their athletes as persons who are likely to provide a source of comfort and security during times of need and who provide a sound platform from which athletes can explore autonomously. A high-quality interdependent coach–athlete relationship is central to effective coaching and is a fundamental precursor of athletes’ optimal functioning [9,12,13]. The benefit of investigating relationships is not for the coaches and athletes exclusively. The need for more research in this area is prompted by the requirements of a systematic, comprehensive, and empirical knowledge, which contains practical implications for coaches, athletes, as well as parents, practitioners, and sport administrators [13]. Jowett et al.’s [14] qualitative case studies and relevant literature were used to generate items for an instrument that measures affective, cognitive, and behavioral aspects of the coach–athlete relationship [15].

Through the Coach–Athlete Relationship Questionnaire (CART-Q), the relationship between coach and athlete has been identified as an important research area for sport psychology [16]. The CART-Q provides an opportunity to pursue research questions that would promote knowledge and understanding of the complex dynamics involved between athletes and coaches from a relationship perspective. It could also contribute to the development of interventions for enhancing the quality of this athletic relationship and its associated outcomes (e.g., motivation, performance, well-being) [17]. The CART-Q [17] includes two versions, namely, direct and meta perspectives, and the investigations and studies that have been conducted focused on both versions; how one person perceives how the other person views him/her (meta-perspective) can dictate/change one’s perception (direct-perspective). The results obtained with the CART-Q can provide relevant information to help professionals in the sport psychology area, and help coaches to develop experiences that promote a positive relationship between coaches and athletes [18]. The relationship between coaches and athletes is crucial for sports performance and individual well-being [19].

The 3Cs model was utilized to understand and investigate the impact of the coach–athlete relationship, namely the constructs of closeness, commitment, and complementarity on the outcomes of the interpersonal relationships. Various studies found associations between the coach–athlete relationship and the outcomes of its interpersonal relationship (e.g., satisfaction, dissatisfaction, anxiety). A study by Jowett and Nezlek [20] aimed to investigate the association between coach–athlete relationship interdependence and satisfaction level as a function of competition level, relationship length, and gender has found that the association between interdependence and satisfaction with training, instruction, and personal treatment are higher within higher-level competitors (e.g., regional, national, and international) rather than lower-level (e.g., club) competitors. The coach–athlete relationship is defined and operationalized as a situation in which both coaches’ and athletes’ feelings of closeness, thoughts of commitment, and behaviors of complementarity (3Cs) are interconnected. Closeness refers to the affective bonds developed between coaches and athletes and includes relational interpersonal properties such as mutual trust and respect. Commitment is defined as an athlete’s and a coach’s intention to maintain a close athletic partnership and long-term relationship that aims to maximize its outcomes. Complementarity refers to the type of interaction that the coach and the athlete engage in that reflects corresponding actions and interactions that are co-operative and affiliative [20].

The Coach–Athlete Relationship Questionnaire (CART-Q) has been validated widely during the last decade. The validation’s attempts crossed the European borders and reached the Far East (China) [21]. Validating the CART-Q began with a study by Jowett and Ntoumanis [15] that consisted of two separate studies. The study aimed to develop an instrument that could be used to assess the nature of the coach–athlete relationship, validate the instrument, and examine the relationships between interpersonal satisfaction and the 3Cs within coaches and athletes. Consequently, the validation studies of the Coach–Athlete Relationship Questionnaire continued targeting different regions, cultures, and countries. Balduck and Jowett [22] examined the psychometric properties of the CART-Q utilizing 144 Belgian coaches. The result supported previous studies that validated this specific instrument, and the psychometric properties of the Belgian version were verified. On the other hand, Yang and Jowett [23] attempted to universalize the CART-Q by conducting a study that recruited participants from seven different countries (United States, Britain, China, Greece, Belgium, Sweden, and Spain). As the results of this study showed a variation within the intensity of the athletes’ perceptions towards the quality of the relationships with their coaches, the validity of the CART-Q was proved using a three-first-order factor model across the seven countries.

Since this important measurement tool was validated in many countries across the world, it is also still unheard of in many other countries. There is still a gap when it comes to investigating the coach–athlete relationship in the Middle East. Therefore, the first aim of this study was to fill that gap and add to the literature by validating this measurement tool; the translated direct and meta-perspective Arabic versions of the CART-Q in the Middle East. Validating the Arabic version of the CART-Q is beneficial to most countries in the Middle East, as most of the countries are Arabic speaking. The CART-Q is highly beneficial to Middle Eastern countries as the sport field in that area is rapidly growing and developing. Many sports events and tournaments take place in the Middle East, as well as the World Cup scheduled to take place in Qatar in 2022, the first-ever World Cup to be held in an Arabic country. Needless to say, many athletes want to pursue professional sport careers in the Middle East, making the coach–athlete relationship a very important relationship to investigate. This leads to emphasizing once again the importance of utilizing and validating the Arabic versions of the CART-Q and helping coaches and athletes to perform at an optimum level. While sport psychology in the Middle East region is underutilized in theoretical, empirical, and practical terms, its various applications may be critical in facilitating harmony and stability in the ways coaches and athletes, as well as other significant individuals, including sport administrators and officials, could operate and interact. The interrelation of both the coach and the athlete makes effective and successful coaching. The wide and various interests from all around the world in exploring the coach–athlete relationship has motivated several sport psychologists to adopt the Coach–Athlete Relationship Questionnaire (CART-Q). It is especially necessary for those who are aiming to develop the sports field in their country. Reaching an optimum level for an athlete can depend on the relationship with her/his coach. As stated, the nature of this relationship includes three important constructs. When these constructs can operationalize well in the relationship of the coach and athlete, the satisfaction level can increase for the athlete. The second aim of this study was to determine the quality of the coach–athlete relationship in relation to athlete’s satisfaction with individual performance. The Athlete Satisfaction Questionnaire (ASQ) is a measure of the experiences of sport participants based on Riemer and Chelladurai’s [24] classification of the facets of athlete satisfaction [25]. Even though research on athlete satisfaction remains limited [26], findings from various studies highlight the importance of satisfaction within the coach–athlete relationship, such as a study by Davis et al. [27], which found that the coach–athlete relationship quality positively predicted athlete satisfaction. Since athlete satisfaction was not fully explored in the Middle East, and since it is highly linked to the coach–athlete relationship, it is very important to discover the facets of athlete satisfaction to predict the quality of the coach–athlete relationship.

This study was conducted in Kuwait, one of the Middle Eastern countries. Satisfaction is very important amongst the Kuwaiti coach–athlete relationships, as satisfaction is a fulfillment of one’s expectations and needs. Once an athlete is fulfilled, it will lead to successful performances and sport results.

## 2. Materials and Methods

### 2.1. Participants

A total of 259 athletes (male and female) were recruited from various athletic clubs in Kuwait. The sample was comprised of 187 male and 72 female athletes, with a mean age of 22 years old (*M*_age_ = 22.33, *SD* = 4.61). The athletes participated in either team or individual sports. Any sport where individuals are organized into opposing teams that compete to win and involves competition between teams of players has been accepted as a team sport. Sport participation type (n_individual_ = 71; n_team_ = 186), sport duration (experience in sport participation) (*M*_year_ = 6.68), and sport achievement (n_yes_ = 123; n_no_ = 134) variables were collected from participants. The participants had to answer a question about their achievements with their current coach. Sport achievement criteria were classified by athletes who won trophies or medals with their current coach and athletes who did not, at the time the questionnaires were conducted. The subjects were extracted randomly. All the participants completed at senior level, and they were members of local clubs.

### 2.2. Measures

#### 2.2.1. The Coach–Athlete Relationship Questionnaire (CART-Q)

CART-Q [17] was used to assess how athletes believe the coach perceives the athletic relationship with them. The questionnaire consists of 11-item meta-perspective items and 3 sub-dimensions: meta-closeness (4 items; e.g., “My coach likes me”), meta-commitment (3 items; e.g., “My coach is committed to me”), and meta-complementarity (4 items; e.g., “My coach is responsive to my efforts during training”). The direct version of the questionnaire also includes 11 direct-perspective items (3 items for direct-closeness; e.g., “I like my coach”; 3 items for direct commitment; e.g., “I am committed to my coach”; 3 items for direct complementarity; e.g., “When I am coached by my coach, I am responsive to his/her efforts”). For this study, the factorial structure of the Arabic version of the CART-Q was used in Kuwait and was prepared with both direct and meta perspectives. The items were assigned a score ranging from 1 (strongly agree) to 7 (strongly disagree) with a mid-point of 4 (half-way). The Cronbach’s internal consistency coefficients for these 3 sub-scales for direct and meta versions were 0.82 and 0.85 for closeness, 0.70 and 0.82 for commitment, 0.65 and 0.82 for complementarity, respectively.

#### 2.2.2. Athlete Satisfaction Questionnaire (ASQ)

ASQ [25] measure the facets of athlete satisfaction. The questionnaire contains 56-items and 15 subscales. These subscales include individual performance, team integration, personal dedication, team performance, ability utilization, strategy, personal treatment, training and instruction, team task contribution, team social contribution, ethics, budget, medical personnel, academic support services, and external agents. In this study, only 3 items were employed to measure athletes’ satisfaction with individual performance (e.g., “I am satisfied with the improvement in my performance over the previous season”). The response scale of these measures ranged from 1 (strongly disagree) to 7 (strongly agree). Cronbach’s alpha reliability estimates ranged from 0.78 to 0.95 [25]. In this study, the internal consistency coefficient for the individual performance subscale was 0.81.

### 2.3. Translation Procedures

The translation and cultural adaptation of the CART-Q was done by six Arabic professionals (three English teachers and three Ph.D. graduates in Physical Education and Sport Science) in Kuwait. Subsequently, the translated items were independently translated back into English. The back-translated version was then compared with the original British version and any inconsistencies, and ways of eliminating them were discussed. This process was repeated until the final versions were accurate and comprehensive translations of the original CART-Q were developed.

### 2.4. Procedures

The approval of the institutional ethics board was not required as per the institution’s guidelines and applicable regulations in the city where the study was conducted. That is, why we cannot provide formal approval by the institutional ethics committee. However, we verified that the study is in accordance with established ethical guidelines. Participation was voluntary, it was not obligatory. All the participants were informed about the anonymity, confidentiality, and the voluntary nature of the study. Before athletes completed forms, written informed consent forms were received from them and from the parents of the athletes who were under the age of 18. Thereby, all potential participants were well-informed about the study. The information stated in the informed consent form was not exceeded. In Kuwait, female athletes are not easy to target since there are very few females participating in sport, and those who do participate are difficult to contact due to their cultural restrictions. An information sheet that explained the aims of the study was also given to the participants. The questionnaires were handed out to the participants and collected at different times. Necessary explanations on how to fill out the questionnaires were given by the first researcher. The questionnaire contained three pages in addition to the demographics section and took 10–15 min to complete.

### 2.5. Data Analysis

The study has two aims. The first aim was to achieve validation of the direct and meta-perspective versions of the CART-Q in the Arabic language, and the second aim was to discover the differentiations of the CART-Q with other variables (e.g., gender, sport participation type, and achievement) using independent sample *t*-tests and to discover the relationships with the variables of age, satisfaction, and sport duration using Pearson correlation coefficients. First of all, all preconditions, which are missing values, normal distribution, and multi-collinearity situations, were checked. A series of descriptive statistics for all variables and sub-dimensions of both meta and direct data were controlled. Mean (*M*), standard deviation (*SD*), skewness, and kurtosis values were obtained to determine whether the data set has a normal distribution. It was seen that skewness and kurtosis values ranged between −0.09 to −2.01. There were no missing values or outliers in the data. Descriptive statistics were calculated for all variables of the study including means, and standard deviations. Multi-collinearity was checked and results showed that tolerance and the variance inflation factor (VIF) value ranges were as they should be. According to Hair, Black, Babin, Anderson, and Tatham [28], the VIF value range is less than 5 and tolerance is more than 0.2.

As the main aim of this study was to examine the psychometric properties of the CART-Q, the first-order confirmatory factor analysis (CFA) was used to confirm the factor structure of the scale. Lisrel 8.1. software package was used to perform the CFA. The covariance matrix was created by using the maximum likelihood calculation method. Furthermore, Pearson correlation was used to examine the relationship of the coach–athlete relationship quality (both direct and meta, separately) with other variables. Independent sample *t*-tests were used to determine the differentiation between gender, sport type, and achievement situation, with direct-meta coach–athlete relationship and satisfaction.

## 3. Results

According to the results obtained from the path analysis with the parameter values for the first level confirmatory factor analysis results (CFA), fit indices (RMSEA: root Mean square error of approximation; CFI: comparative fit index; GFI: goodness of fit index; and AGFI: adjusted goodness of fit index) of the data collected by direct-method were as follows (x^2^/df = 2.21; RMSEA = 0.06; CFI = 0.98; GFI = 0.95; AGFI = 0.91); data for the meta-method were as follows (x^2^/df = 2.32; RMSEA = 0.08; CFI = 0.99; GFI = 0.93; AGFI = 0.87). According to acceptable criteria, fit index values should be between <2–3 for x^2^/df; <0.05–0.10 for RMSEA; <0.95–0.97 for CFI; <0.90–0.95 for GFI; and <0.90–0.95 for AGFI [29]. Results show that the results obtained for both scales are compatible with the model data and are acceptable. These results show that the data obtained from the research corresponds to the predicted theoretical structure of the CART-Q versions for Kuwait. On the other hand, when interpreting the CFA, Lambda (factor loading), t, and R^2^ values of the substances are also important (Table 1 and Table 2).

As seen in Table 1, Lambda (λ), t, and R^2^ values obtained as significant at 0.05 level. When lambda values showing factor loads are examined, it is seen that factor loads vary between 0.32 and 0.87. These values show that the factor loadings of the items are acceptable. In addition, t values of the observed variables (items) regarding the explanation of latent variables (dimensions) were found to be significant at the 0.05 level. On the other hand, when R^2^ values are analyzed, it is seen that the variance amount explained by the sub-factors in the items varies between 0.11 and 0.74. All these findings can be considered as evidence that the scale has satisfactory construct validity.

Lambda (λ), t, and R^2^ values obtained in Table 2 are significant at the 0.05 level. When lambda values showing factor loads are examined, it is seen that factor loads vary between 0.67 and 0.88. These values show that the factor loadings of the items are acceptable. Besides, t values of the observed variables (items) regarding the explanation of latent variables (dimensions) were found to be significant at 0.05 level. On the other hand, when R^2^ values are analyzed, it is seen that the variance amount, explained by the sub-factors in the items, varies between 0.44 and 0.78. All these findings can be considered as evidence that the scale has satisfactory construct validity.

Means and standard deviations of all subscales in the study concerning gender, sports participation type, and achievement situation are presented in Table 3.

Table 3 shows the means and standard deviations of all sub-scales concerning gender, sports participation type, and achievement situation. According to this, both females and males with achievement had higher means than participants without achievement. Furthermore, individual sports participants for both female and male, with achievement, had higher means than team sport participants without achievement.

As seen in Table 4, there was a positive correlation between age and sport duration (r = 0.41, *p* < 0.01). Commitment (r = 0.16, *p* < 0.05) and complementarity (r = 0.17, *p* < 0.01) were positively associated with age; while any subscale was significantly associated with sport duration. Furthermore, satisfaction was positively correlated with closeness (r = 0.24, *p* < 0.01), commitment (r = 0.33, *p* < 0.01), and complementarity (r = 0.28, *p* < 0.01). Additionally, all subscales were positively associated with itself. According to these findings, commitment and complementarity scores increased with increasing age; however, it was found that there was no difference between the duration of sports and any sub-dimension.

As seen in Table 5, there was a positive correlation between age and sport duration (r = 0.41, *p* < 0.01). Meta-commitment (r = 0.14, *p* < 0.05) and meta-complementarity (r = 0.14, *p* < 0.05) were positively associated with age; while sport duration was significantly associated with meta-closeness (r = 0.16, *p* < 0.05) and meta-commitment (r = 0.14, *p* < 0.05). Furthermore, satisfaction was positively correlated again with meta-closeness (r = 0.29, *p* < 0.01), meta-commitment (r = 0.33, *p* < 0.01), and meta-complementarity (r = 0.30, *p* < 0.01). Additionally, all subscales were positively associated with itself. According to these results, meta-commitment and meta-complementarity scores increased with increasing age; it was also found that as the duration of sports increased, meta-closeness and meta-commitment scores increased. The scores of the athletes on satisfaction and the CART-Q (both meta and direct) scales were examined according to gender.

The independent sample *t*-test results were presented in Table 6. According to the findings, significant differences were found in terms of satisfaction and CART-Q scores according to gender. In all sub-dimensions, the means of female athletes were significantly higher than the means of male athletes.

According to the findings in Table 7, significant differences were found in favor of individual participants in some dimensions according to the sport participation type. Accordingly, the means obtained were commitment (*M*_individual_ = 5.99; *SD* = 0.99; t (255) = 2.33; *p* < 0.05), meta-closeness (*M*_individual_ = 6.31; *SD* = 0.81; t (255) = 3.45 *p* < 0.01), meta-commitment (*M*_individual_ = 5.94; *SD* = 1.02; t (255) = 3.20; *p* < 0.01), and meta-complementarity (*M*_individual_ = 6.20; *SD* = 0.94; t (255) = 2.84; *p* < 0.01). In all of the dimensions where there is a significant difference, the means of athletes who participated in individual sports were higher than the means of athletes who participated in team sports. These results showed that the perceived relationship levels of the athletes who participated in individual sports with their coaches were higher than the athletes who participated in team sports.

Regarding the findings that represented in Table 8, significant differences were obtained in all satisfaction and CART-Q scores according to the achievement status of the athletes. The means of the athletes having success in all sub-dimensions were higher than the athletes who did not. These results showed that the coach–athlete relationship quality scores of the athletes with achievement are higher than the athletes without achievement.

## 4. Discussion

The development of the Coach–Athlete Relationship Questionnaire (CART-Q) was guided by the findings of previous qualitative studies, and the development of the British [15], the Greek [30], and the Chinese [21] versions of the CART-Q. The CART-Q is expanding around the world and its validation is evidenced across the globe, despite its linguistic differences, such as in Brazil [18], China [21], and Belgium [22]. The CART-Q has become an important measurement tool and it is time to develop and utilize the CART-Q in new regions, such as the Middle East. New versions of a translated CART-Q to Arabic, Persian, and other languages are needed. These areas have different cultures, traditions, and beliefs, which make it necessary and encouraging to explore the coach–athlete relationship in such regions. The results of the CART-Q could help researchers and practitioners who aim to investigate the nature of the coach–athlete relationship in the Middle East or among the Arab nations. This study shows robust evidence of the validity of the direct and meta-perspectives of the Arabic version of the CART-Q. Moreover, the CART-Q could help coaches and athletes from the Middle East to understand the reasons and methods that lead to a quality coach–athlete relationship.

The first aim of this present study was to examine the validity of the direct and meta-perspective versions of the Coach–Athlete Relationship Questionnaire (CART-Q) in the Middle East area (Kuwait) and to discover the differentiations of the CART-Q with other variables. An understanding of the nature and content of the coach–athlete relationship, as well as its functions, is important because such knowledge could contribute toward the development of strategies that help to establish and maintain effective and successful coach–athlete relationships [13,31]. Confirmatory factor analysis (CFA) was used to examine the validity of the Arabic CART-Q version, which is consistent with a study by Vieira et al., [18] which confirmed the construct validity of the Brazilian coach–athlete questionnaire by using CFA. The methodological procedures that were used in this study to examine the factorial validity of the direct and meta-perspective versions of the Arabic CART-Q are coupled with the procedures in previous studies [15,17,23]. When the factorial construct was examined for Arabic culture, the factor loadings of direct complementarity were seen lower than other dimensions. This result can be caused by Arabic culture than itself because complementarity reflects the members’ reciprocal and corresponding cooperation [32]. In Arabic culture, considering the lower number of female participants in the sport field, this result may make sense more. Furthermore, complementarity has been studied as an issue [33] across cultures, because the nature of the attitude of friendly and relaxed can be differentiated in a different culture. To avoid incomplete implications, measurement invariance analyses are recommended for the future.

The second aim of this present study was to determine the quality of the coach–athlete relationship to athlete satisfaction. Other researchers such as Jowett et al. [34], approached this relationship similarly and it showed that athletes who feel that their relationship with the coach is underlined by trust, respect, and co-operation, are more likely to be satisfied. High satisfaction leads to a high-quality coach–athlete relationship, and the coach–athlete relationship quality positively predicts athlete satisfaction [27].

The scores of the athletes on satisfaction and the CART-Q (both meta and direct) scales were examined according to gender. According to the findings, significant differences were found in terms of satisfaction and CART-Q scores according to gender. In all sub-dimensions, the means of female athletes were significantly higher than the means of male athletes. It can be presumed that these results are because in Kuwait, the cultural norm is for female athletes to have female coaches. This gives the female athletes a sense of satisfaction within their sport environment. Every culture has its unique norms, values, and beliefs, however, less attention has been given to how culture affects the coach–athlete relationship. Within the sport psychology literature, it has been noted that there is less attention paid to the cultural impact and the cultural background effect [35].

Females are more emotional, caring, understanding of one another, and they structurally have more intense connectedness and trust needs than men [36], therefore, the female athletes tend to be committed to their coaches. However, in Kuwait, the media is a male-dominated setting where women’s sport is rarely broadcasted and female athletes do not get the attention they deserve. Therefore, once females become athletes, they try to prove that they deserve the same attention as men, giving importance to their sports career, showing their athletic abilities, and committing to their coaches. This level of commitment makes the sport environment a safe and satisfactory place for female athletes. Females have a very strong ability to connect, and the female coaches have the right strategies to create a comfort zone for their female athletes. These findings were similar to what was found in the study by Jowett and Nezlek [20], where it was mentioned that “all-female dyads were more satisfied with training and instruction than the other gender combinations considered together” [20]. The findings of this study show that the same gender coach–athlete dyads make a successful combination, and are consistent with a study by Jowett [37], where it was found that the same gender coach–athlete dyads may feel they have something in common that connects them.

This reflects a high degree of commitment between dyads and a desire to maintain a long-term sport relationship. The dyads invest in each other and are committed to each other. These results are consistent with the study by Jowett and Nezlek [20], as it was found that coaches invest their knowledge, skills, and expertise, while athletes invest their raw talent, long and hard hours of training, passion, determination, and motivation to achieve.

The results also showed that the perceived relationship levels of the athletes who participated in individual sports with their coaches were higher than the athletes who participated in team sports. The coach–athlete relationship is important in individual sports, as the leadership and orientation of the coach are very important for the athletes who are in direct contact with them [36]. Baker et al., [38] found that team sport athletes require greater coach control, and when those needs were not met, it created less satisfaction in both the athlete and the coach in team sports. Significant differences were obtained in all satisfaction and CART-Q scores according to the achievement status of the athletes. The means of the athletes having success in all sub-dimensions were higher than the athletes who did not. These results showed that the coach–athlete relationship quality scores of the athletes with achievement are higher than the athletes without achievement. Moreover, coaches’ satisfaction is likely to be linked to their own and their athletes’ perspectives of the coach–athlete relationship quality, while athletes’ satisfaction is likely to be associated with their meta-perspectives and their coaches’ meta-perspectives of the relationship quality [39].

The findings of this study revealed that there was a positive correlation between age and sport duration and no difference between the duration of sports and any sub-dimension. According to the results, older athletes had higher commitment and complementarity. It was observed that as the sport duration increased, meta-closeness and meta-commitment increased. This study shows evidence of the validity of the direct and meta-perspective Arabic versions of the CART-Q. These results could help researchers and practitioners who aim to investigate the nature of the coach–athlete relationship in the Arabic speaking nations in the Middle East. Moreover, the results of the present study could help coaches and athletes from the Middle East to understand the reasons and methods that lead to a quality coach-athlete relationship.

The present study includes also some limitations that needed to be explained. Firstly, the sample was taken for this study only targeted athletes and therefore did not include a full dyad. It would have been more beneficial to target coaches in the study, however, in Kuwait, there is a very large number of foreign coaches. Getting the foreign, non-Arabic speaking coaches involved in this study would require the coaches to use the original (English) version of the CART-Q, and that would go against the purpose of this study, as this study requires the use of the Arabic version of the CART-Q. Secondly, there are very few female athletes in the sample as it is not popular to find female athletes in Kuwait due to the cultural differences that discourage the existence of women in the sports field. That is why the results should be interpreted with caution as the sample sizes are significantly different (gender and sport type). Thirdly, at the time this study was conducted, Kuwait was banned from international sport competitions. As a result, athletes in Kuwait were significantly affected in terms of their commitment, and motivation. Most of the athletes lacked ambition, had fewer training hours, and had less desire to improve or prepare for a sports career. In turn, they were frustrated and disappointed. This situation could have greatly affected the results of this study. In the future, it would be highly beneficial to investigate such issues and the impact they have on the coach–athlete relationship.

## 5. Conclusions

The results of the Arabic version of the CART-Q could help researchers and practitioners investigate the nature of the coach–athlete relationship in the Middle East or among the Arab nations. Furthermore, the CART-Q could help coaches and athletes understand the steps that lead to a quality coach–athlete relationship while investigating the obstacles that stand in the way of achieving a harmonious relationship. Once coaches and athletes understand what builds up their relationship, they can achieve their goals together and reach a state of overall well-being. The constructs of the CART-Q have shown that high satisfaction is strongly associated with a high quality, successful, harmonious, and supportive coach–athlete relationship [17]. The CART-Q is highly beneficial to Middle Eastern countries as the sports field in that area is rapidly growing and developing.

## Figures and Tables

**Table 1:** According to The Coach–Athlete Relationship Questionnaire (CART-Q)’s confirmatory factor analysis (CFA) results, standardized Lambda (λ), t, and R^2^ values for the direct method.

Subscales and Items	λ	t	R^2^
Closeness 1	0.87	17.17 *	0.74
Closeness 2	0.80	15.42 *	0.67
Closeness 3	0.58	9.62 *	0.33
Closeness 4	0.74	13.71 *	0.54
Commitment 1	0.69	12.43 *	0.47
Commitment 2	0.48	7.79 *	0.23
Commitment 3	0.83	15.93 *	0.70
Complementarity 1	0.88	16.97 *	0.74
Complementarity 2	0.32	5.31 *	0.11
Complementarity 3	0.46	7.68 *	0.23
Complementarity 4	0.52	8.95 *	0.28

Note. * *p* < 0.05.

**Table 2:** According to CART-Q’s CFA results, standardized Lambda (λ), t, and R^2^ values for the meta-method.

Subscales and Items	λ	t	R^2^
Meta-Closeness 1	0.82	15.98 *	0.67
Meta-Closeness 2	0.78	14.87 *	0.60
Meta-Closeness 3	0.67	11.97 *	0.45
Meta-Closeness 4	0.88	18.13 *	0.78
Meta-Commitment 1	0.82	15.95 *	0.67
Meta-Commitment 2	0.78	14.94 *	0.61
Meta-Commitment 3	0.74	13.83 *	0.55
Meta-Complementarity 1	0.74	13.98 *	0.55
Meta-Complementarity 2	0.67	12.08 *	0.44
Meta-Complementarity 3	0.82	16.08 *	0.67
Meta-Complementarity 4	0.72	13.26 *	0.51

Note. * *p* < 0.05.

**Table 3:** Means and standard deviations of all sub-scales concerning gender, sport participation type (SPT), and achievement situation.

Gender	SPT	Achievement	*M* ± *SD*	Closeness	Commitment	Complementarity	Meta-Closeness	Meta-Commitment	Meta-Complementarity	Satisfaction
Female	Individual	No	*M*	6.15	6.03	6.27	6.35	5.93	6.15	5.51
			*SD*	0.96	1.23	0.84	0.88	1.11	1.26	2.10
		Yes	*M*	6.76	6.44	6.56	6.80	6.28	6.62	6.13
			*SD*	0.30	0.46	0.46	0.30	0.64	0.47	0.75
		Total	*M*	6.52	6.28	6.44	6.62	6.14	6.43	5.88
			*SD*	0.70	0.85	0.64	0.62	0.86	0.88	1.44
	Team	No	*M*	6.10	5.41	6.37	5.43	5.16	5.96	5.77
			*SD*	0.84	0.93	0.47	1.15	1.24	0.80	1.23
		Yes	*M*	6.49	6.13	6.45	6.45	6.24	6.38	6.00
			*SD*	0.64	.88	0.67	0.73	0.93	0.73	1.07
		Total	*M*	6.36	5.89	6.43	6.11	5.87	6.24	5.92
			*SD*	0.73	0.95	0.61	1.01	1.15	0.77	1.12
	Total	No	*M*	6.12	5.65	6.33	5.78	5.46	6.03	5.67
			*SD*	0.87	1.08	0.63	1.13	1.23	0.98	1.59
		Yes	*M*	6.58	6.23	6.49	6.56	6.26	6.46	6.04
			*SD*	0.56	0.78	0.61	0.64	0.84	0.66	0.97
		Total	*M*	6.41	6.02	6.43	6.28	5.97	6.31	5.91
			*SD*	0.72	0.93	0.61	0.92	1.06	0.814	1.23
Male	Individual	No	*M*	5.50	5.11	5.75	5.36	4.88	5.19	5.11
			*SD*	1.29	1.09	0.99	1.21	1.13	1.13	1.77
		Yes	*M*	6.35	6.00	6.28	6.34	6.05	6.30	5.55
			*SD*	0.80	0.95	0.64	0.63	0.96	0.77	1.32
		Total	*M*	6.18	5.83	6.17	6.15	5.82	6.08	5.46
			*SD*	0.96	1.03	0.74	.858	1.08	0.953	1.41
	Team	No	*M*	5.88	5.46	5.95	5.60	5.04	5.49	5.20
			*SD*	1.22	1.31	0.941	1.38	1.49	1.33	1.30
		Yes	*M*	6.25	5.74	6.08	6.10	5.72	5.94	5.35
			*SD*	1.01	1.01	0.82	0.87	1.05	1.00	1.37
		Total	*M*	6.06	5.63	6.04	5.87	5.42	5.76	5.31
			*SD*	1.11	1.17	0.864	1.16	1.33	1.18	1.34
Total		No	*M*	5.85	5.43	5.93	5.57	5.02	5.47	5.19
			*SD*	1.23	1.29	0.94	1.36	1.46	1.31	1.34
		Yes	*M*	6.29	5.85	6.16	6.20	5.86	6.09	5.43
			*SD*	0.92	0.98	0.75	0.78	1.02	0.92	1.35
		Total	*M*	6.06	5.63	6.04	5.87	5.42	5.76	5.31
			*SD*	1.11	1.17	0.86	1.16	1.33	1.18	1.34

Note. *M*: Mean; *SD*: Standard deviation.

**Table 4:** Correlation coefficients among age, sport duration, satisfaction, and direct data.

Variables	1	2	3	4	5	6
1. Age	1					
2. Sport Duration	0.41 **	1				
3. Satisfaction	0.03	0.01	1			
4. Closeness	0.07	0.02	0.24 **	1		
5. Commitment	0.16 *	0.07	0.33 **	0.72 **	1	
6. Complementarity	0.17 **	0.03	0.28 **	0.70 **	0.77 **	1

Note. * *p* < 0.05, ** *p* < 0.01.

**Table 5:** Correlation coefficients among age, sport duration, satisfaction, and metadata.

Variables	1	2	3	4	5	6
1. Age	1					
2. Sport Duration	0.41 **	1				
3. Satisfaction	0.03	0.01	1			
4. Meta-closeness	0.09	0.16 *	0.29 **	1		
5. Meta-commitment	0.14 *	0.14 *	0.33 **	0.84 **	1	
6.Meta-complementarity	0.14 *	0.09	0.30 **	0.85 **	0.87 **	1

Note. * *p* < 0.05, ** *p* < 0.01.

**Table 6:** Independent sample *t*-test results for gender and each scale.

	Gender	*n*	*M*	*SD*	df	t	*p*
Satisfaction	Female	72	5.91	1.23	255	3.26 ***	0.001
	Male	185	5.31	1.34			
Closeness	Female	72	6.41	0.72	255	2.97 **	0.003
	Male	185	6.06	1.11			
Commitment	Female	72	6.02	0.94	255	2.79 **	0.006
	Male	185	5.63	1.17			
Complementarity	Female	72	6.43	0.62	255	4.05 ***	0.000
	Male	185	6.04	0.86			
Meta-Closeness	Female	72	6.28	0.93	255	2.68 **	0.008
	Male	185	5.87	1.16			
Meta-Commitment	Female	72	5.97	1.06	255	3.11 **	0.002
	Male	185	5.42	1.33			
Meta-Complementarity	Female	72	6.31	0.81	255	3.58 ***	0.000
	Male	185	5.76	1.18			

Note. ** *p* < 0.01; *** *p* < 0.001; *M*: Mean; *SD*: Standard deviation; df: Degrees of freedom.

**Table 7:** Independent sample *t*-test results for sport participation type (SPT) and each scale.

	SPT	*n*	*M*	*SD*	df	t	*p*
Satisfaction	Individual	71	5.61	1.42	255	0.99	0.325
	Team	186	5.42	1.30			
Closeness	Individual	71	6.30	0.89	255	1.46	0.182
	Team	186	6.11	1.07			
Commitment	Individual	71	5.99	0.99	255	2.33 *	0.021
	Team	186	5.65	1.16			
Complementarity	Individual	71	6.27	0.72	255	1.54	0.156
	Team	186	6.10	0.86			
Meta-Closeness	Individual	71	6.31	0.81	255	3.45 **	0.004
	Team	186	5.86	1.19			
Meta-Commitment	Individual	71	5.94	1.02	255	3.20 **	0.005
	Team	186	5.44	1.35			
Meta-Complementarity	Individual	71	6.20	0.94	255	2.84 **	0.010
	Team	186	5.80	1.16			

Note. * *p* < 0.05, ** *p* < 0.01; *M*: Mean; *SD*: Standard deviation; df: Degrees of freedom.

**Table 8:** Independent sample *t*-test results for achievement and each scale.

	Achievement	*n*	*M*	*SD*	df	t	*p*
Satisfaction	No	123	5.29	1.40	255	−2.09 *	0.038
	Yes	134	5.64	1.26			
Closeness	No	123	5.91	1.16	255	−3.80 ***	0.000
	Yes	134	6.39	0.83			
Commitment	No	123	5.48	1.25	255	−3.64 ***	0.000
	Yes	134	5.98	0.94			
Complementarity	No	123	6.01	0.89	255	−2.53 **	0.012
	Yes	134	6.27	0.72			
Meta-Closeness	No	123	5.62	1.31	255	−5.24 ***	0.000
	Yes	134	6.33	0.76			
Meta-Commitment	No	123	5.11	1.42	255	−5.74 ***	0.000
	Yes	134	6.00	0.98			
Meta-Complementarity	No	123	5.59	1.26	255	−4.63 ***	0.000
	Yes	134	6.22	0.86			

Note. * *p* < 0.05; ** *p* < 0.01; *** *p* < 0.001; *M*: Mean; *SD*: Standard deviation; df: Degrees of freedom.

## Data Availability

The data presented in this study are available on request from the corresponding author.
